# Premature Senescence and Increased Oxidative Stress in the Thymus of Down Syndrome Patients

**DOI:** 10.3389/fimmu.2021.669893

**Published:** 2021-06-01

**Authors:** Genni Enza Marcovecchio, Francesca Ferrua, Elena Fontana, Stefano Beretta, Marco Genua, Ileana Bortolomai, Anastasia Conti, Davide Montin, Maria Teresa Cascarano, Sonia Bergante, Veronica D’Oria, Alessandro Giamberti, Donato Amodio, Caterina Cancrini, Adriano Carotti, Raffaella Di Micco, Ivan Merelli, Marita Bosticardo, Anna Villa

**Affiliations:** ^1^ San Raffaele Telethon Institute for Gene Therapy (SR-TIGET), IRCCS San Raffaele Scientific Institute, Milan, Italy; ^2^ Paediatric Immunohematology and Bone Marrow Transplantation Unit, IRCCS San Raffaele Scientific Institute, Milan, Italy; ^3^ Vita-Salute San Raffaele University, Milan, Italy; ^4^ Humanitas Clinical and Research Center, Rozzano, Italy; ^5^ Milan Unit, Istituto di Ricerca Genetica e Biomedica (IRGB), Consiglio Nazionale delle Ricerche (CNR), Milan, Italy; ^6^ Department of Pediatric and Public Health Sciences, University of Torino, Turin, Italy; ^7^ Regina Margherita Children’s Hospital, AOU Città della Salute e della Scienza di Torino, Turin, Italy; ^8^ Cardiochirurgia Pediatrica Ospedale Infantile Regina Margherita (OIRM), AOU Città della Salute e della Scienza di Torino, Turin, Italy; ^9^ Laboratory of Stem Cells for Tissue Engineering, Istituto di Ricovero e Cura a Carattere Scientifico, Policlinico San Donato, Milan, Italy; ^10^ Department of Pediatric Cardiac Surgery, IRCCS San Donato Milanese Hospital, San Donato Milanese, Italy; ^11^ Department of Congenital Cardiac Surgery, IRCCS Policlinico San Donato, San Donato Milanese, Italy; ^12^ Department of Systems Medicine, University of Rome Tor Vergata, Rome, Italy; ^13^ University Department of Pediatrics, Bambino Gesù Children’s Hospital, IRCCS, Rome, Italy; ^14^ Department of Pediatric Cardiac Surgery, IRCCS Bambino Gesú Children’s Hospital, Rome, Italy; ^15^ Institute for Biomedical Technologies-National Research Council, Segrate, Italy; ^16^ Laboratory of Clinical Immunology and Microbiology, IDGS, DIR, NIAID, NIH, Bethesda, MD, United States

**Keywords:** Down syndrome, thymus, thymic epithelial cells, senescence, oxidative stress

## Abstract

Down syndrome (DS) patients prematurely show clinical manifestations usually associated with aging. Their immune system declines earlier than healthy individuals, leading to increased susceptibility to infections and higher incidence of autoimmune phenomena. Clinical features of accelerated aging indicate that trisomy 21 increases the biological age of tissues. Based on previous studies suggesting immune senescence in DS, we hypothesized that induction of cellular senescence may contribute to early thymic involution and immune dysregulation. Immunohistochemical analysis of thymic tissue showed signs of accelerated thymic aging in DS patients, normally seen in older healthy subjects. Moreover, our whole transcriptomic analysis on human Epcam-enriched thymic epithelial cells (hTEC), isolated from three DS children, which revealed disease-specific transcriptomic alterations. Gene set enrichment analysis (GSEA) of DS TEC revealed an enrichment in genes involved in cellular response to stress, epigenetic histone DNA modifications and senescence. Analysis of senescent markers and oxidative stress in hTEC and thymocytes confirmed these findings. We detected senescence features in DS TEC, thymocytes and peripheral T cells, such as increased β-galactosidase activity, increased levels of the cell cycle inhibitor p16, telomere length and integrity markers and increased levels of reactive oxygen species (ROS), all factors contributing to cellular damage. In conclusion, our findings support the key role of cellular senescence in the pathogenesis of immune defect in DS while adding new players, such as epigenetic regulation and increased oxidative stress, to the pathogenesis of immune dysregulation.

## Introduction

Down syndrome (DS) is the most common chromosomal anomaly among live-born infants, typically characterized by complete or partial trisomy of chromosome 21 (Chr21) [OMIM#190685] (1). Its incidence ranges between 1:100 and 1:1000 live births in general population and is influenced by maternal age (2, 3). DS is one of the most common genetic cause of intellectual disability (4) and its complex phenotype results from a dosage imbalance of genes located on human Chr21. In addition to learning disabilities, there are various common features occurring in all DS patients, such as craniofacial abnormalities and hypotonia in early infancy (5). Congenital heart disease (CHD) is regarded as one of the most important clinical phenomena in children with DS, due to its significant impact on morbidity and mortality (6). DS is also associated with a group of clinical manifestations of accelerated aging (7).

Previous studies in *Ts65Dn* mice, a well-characterized mouse model of DS, have evidenced defects in hematopoietic progenitor cell development and function at the level of both hematopoietic stem cells (HSC) and lymphoid progenitors, which have thymus-seeding potential, with increased oxidative stress and decreased IL7Rα expression as indicated causes of these alterations (8). Further studies in *Ts65Dn* mice have also shown a decrease in the number and proportion of immature, double negative (DN) thymocytes, double positive (DP) and single positive (SP) CD4 thymocytes (9). Similarly to lymphoid progenitors, a reduced expression of IL7Rα was also detected in immature thymocyte subsets, likely mediated by higher oxidative stress and Notch pathway inhibition (9). A senescent phenotype was suggested by reduced naïve T cells in the spleen and reduced proliferation to polyclonal stimulation of peripheral T cells (8).

Several studies indicated alterations in the thymic stroma and in the thymocytes of DS patients, with defects in both immature progenitor cells and mature peripheral lymphocytes (10, 11). Early reports showed that the thymus is smaller in DS subjects, with an abnormal structure showing signs of premature thymic involution, with loss of cortico-medullary demarcation and markedly enlarged Hassall’s corpuscles (12). Our previous study confirmed the significant weight reduction of DS thymi as compared to age-matched HDs (11). Additionally, the thymic tissue from the DS patients that we analyzed showed an accelerated maturation of the thymic epithelial compartment, with signs of premature involution (11). Increased frequency of peripheral γδ-T cells and lower frequency of naïve T cells have also been reported (13). Decreased numbers of recent thymic emigrant (RTE) cells (13) and significantly lower TREC levels have been observed, suggesting decreased thymopoiesis (11–14). Expression of thymic-specific proteasome subunit β5t, but not of cathepsin, has also been reported as markedly reduced in DS thymi (15). These findings suggest that abnormal thymic architecture and decreased expression of functionally important molecules in thymic stroma may contribute to altered thymic function and constitute a causative factor for immunological abnormalities in DS patients. Impairment of thymic function in DS patients could indeed explain their higher risk to develop autoimmune phenomena, as compared to age-matched individuals. Impaired function of natural T regulatory cells (nTreg), generated in the thymus, has been shown in several reports on DS patients (16). Of note, we showed that, although Treg cells were higher in number both in the periphery and in thymus, they were impaired in their suppressive ability (11), suggesting that the profound anatomical and architectural abnormalities of DS thymus may affect nTreg cell functionality.

To the best of our knowledge, no studies focused on TEC of DS patients have been reported to date. Indeed, published gene expression analyses have been performed on the whole thymus without distinguishing the epithelial component and thymocytes (16, 17). Two studies reported that the expression of *AIRE* gene, located on 21q22.3, is reduced in DS thymus, as compared to age-matched controls, leading to global thymic hypofunction and central tolerance failure (17, 18). Remarkably, we detected a statistically significant increased expression of AIRE and Ins2, a tissue-restricted antigen (TRA) induced by AIRE, in DS thymic tissue of children under one-year of age, when compared to age-matched controls (11). These results are in contrast with what we noticed in DS children aged between 2 and 5 years, in which we detected a remarkable decrease in AIRE expression, which correlates well with the presence of large cystic involutions, a sign of premature aging, and decreased thymopoietic activity (11).

Transcriptome analysis of the whole thymus revealed contrasting data, showing down regulation of genes involved in antigen processing and presentation and in thymic T-cell differentiation/selection, as well as downregulation of TRAs (18), while another study performed on thymocyte-depleted thymic specimens showed elevated expression of AIRE mRNA and a trend toward increased expression of some AIRE-dependent TRA genes in DS patients (19). Increased frequency of AIRE^+^ mTEC and CD11c^+^ DC and enlarged Hassall’s corpuscles were also showed, as part of altered cell composition and architecture of thymic medulla in these patients (19). A recent study evaluated the impact of trisomy 21 on thymic gene interaction networks, through gene co-expression network and miRNA-target analyses and showed that epigenetic mechanisms acting at chromatin level and through the miRNA control of transcriptional programs involving the networks high-hierarchy genes contribute to thymic tissue adaptation occurring in trisomy 21 genomic dysregulation (20). We also detected alterations in the kinetics of DS thymocytes differentiation with a skewing towards increased thymocyte maturation (11). Altogether these results support central tolerance perturbation in DS patients contributing to increased susceptibility to develop autoimmune signs.

To further dissect the contribution of the epithelial and the lymphoid component to the increased susceptibility to develop immunodeficiency and immune dysregulation, we set out to study thymic tissue removed from pediatric DS patients undergoing cardiac surgery. We performed immunohistochemistry studies on the thymic tissue and transcriptomic analysis on EpCam-enriched cells. We then validated transcriptome-specific alterations found in DS patients in TEC, thymocytes and peripheral T cells.

## Materials and Methods

### Human Thymic Specimen Collection

In order to collect human post-natal thymic specimens, collaborations with the Pediatric heart surgery departments of “Bambino Gesù” Children’s Hospital (Rome), Policlinico San Donato (Milan) and Regina Margherita Children’s Hospital (Turin) were established. The required protocols for biological sample collection for research purpose and relative informed consents were prepared and approved by local Ethical Committees (DGS_Project_OPBG_2015 and TIGET07 protocol). Parents or legal guardians of enrolled subjects signed the informed consent forms. Enrolled patients were either pediatric patients affected by DS, or age-matched children with congenital heart disease (CHD) without known immune defects (control group), undergoing heart surgery for the first time with median sternotomy. Patients affected by a known infectious or immune system disease and characterized by a previous history of chemo/radiotherapy were excluded from the study. Thymic samples were collected during heart surgery, during which thymic tissue is usually removed, entirely or partially, to access the operating field. After surgery, thymic specimens were kept in normal saline solution at +4°C and processed within 24-48 hours.

### Human PBMC Collection

Peripheral blood of HD and DS patients were obtained according to The Code of Ethics of the World Medical Association (Declaration of Helsinki) with the approval of the local Ethical Committees of the Policlinico San Donato and San Raffaele Scientific Institute (TIGET07).

In our study we enrolled 13 Healthy Donors (HD) and 12 DS patients both undergoing cardiac surgery. To increase the number of DS patients included in the study, we collected peripheral blood samples from the Pediatric Department of San Raffaele Hospital. Samples were obtained according to the Helsinki Declaration with the approval of the local Medical Ethical Committees of the San Raffaele Scientific Institute Internal Review Board (TIGET06). Written informed consent was obtained from parents and/or legal guardians for sample collection. A description of all HDs and patients is reported in **Supplemental Tables 1–3**, respectively. In our study patients have been divided in different age groups, known to differentiate as regards thymic maturational processes, which were determined based on previous reports (21) (**Supplemental Table 4**).

### Thymic Tissue Processing: Thymocyte Recovery and htec Isolation and Enrichment

After arrival at SR-Tiget, thymic tissue was cleaned from blood vessels, clots and surrounding fat and connective tissue, weighted and cut in small pieces. About 1 gram of tissue was then collected and fixed in formalin for histological studies. Thymocytes were recovered by mashing thymic fragments with a sterile syringe plunger. Supernatant containing released thymocytes was removed and replaced with fresh PBS. This was repeated until the supernatant became relatively transparent. All these steps were performed on ice. Thymocytes were kept on ice in PBS (CORNING, Corning, NY, USA) containing 1% of penicillin/streptomycin (P/S) (ThermoFisher scientific, Waltham, Massachusetts, USA) and 10% FBS (Sigma-Aldrich, Saint Louis, Missouri, USA) to preserve their viability.

hTEC isolation was performed using a published protocol (22), further optimized by our group. Thymic samples were digested at 37°C with a solution containing Liberase TL (Roche, Basel, Switzerland) and DNase I (Sigma-Aldrich, Saint Louis, Missouri, USA) in 3 steps of 40, 40 and 30 minutes of length, respectively. After each digestion step, supernatant was collected and kept at 37°C, upon addition of an equal volume of RPMI (CORNING, Corning, NY) containing 10% FBS and 1% P/S. At the end of the whole digestion process, all 3 fractions were pooled and centrifuged at 1,500 rpm for 5 min. The thymic single cell suspension was then incubated for 15 minutes at +4°C with anti-human CD45 microbeads (Miltenyi Biotec, Bergisch Gladbach, Germany) and then processed with the autoMACS Pro Separator (Miltenyi Biotec). The CD45-negative fraction, enriched in thymic stromal cells and depleted from hematopoietic cells, was retrieved and then tested by multicolor FACS analyses for the expression of TEC markers or processed for further sorting.

### Sorting of Human TEC

Human TEC (hTEC) sorting was performed on freshly digested thymic stromal cells or after thawing human thymic samples, previously frozen soon after digestion, and after CD45+ cell-depletion with AutoMACS Pro Separator. Briefly, cells were stained with the following antibodies: anti-CD45 VioBlue (clone 5B1), anti-Epcam PeVio770 (clone HEA-125), anti-CD31 APCVio770 (AC128), anti-HLA-DR, DP, DQ APC (REA332) (all from Miltenyi Biotec), anti-Ulex-1 FITC (FL 1061, Vector), and anti-CD205 PE (HD83, Biolegend) antibodies and sorted with a FACSAria Fusion (Becton Dickinson) cell sorter (FRACTAL facility of San Raffaele Hospital, Milan, Italy), with a 85 micron nozzle. Non-viable cells were excluded from analyses using 7-AAD (BD Pharmingen). Cells were sorted directly in lysis buffer (LB) for next RNA extraction. Lysis buffer from “ReliaPrepTM RNA Cell Miniprep system” kit was used. 1-thioglicerol (TG) was added to LB at a 1:100 dilution, according to manufacturer’s instructions. Sorted cells in LB+TG were then stored at -80° until use.

### RNA-Seq

RNA was extracted from Epcam-enriched hTEC subsets by using ReliaPrepTM RNA MiniPrep System (Promega) according to manufacturer’s recommendations. RNA was then stored at -80°C until use. Full-length RNA-seq libraries were prepared using the SMART-Seq2 protocol (23), with minor modification. Briefly, RNA (1–5 ng) was reverse transcribed using custom oligodT and template-switching LNA oligos (sequences), followed by PCR amplification and clean-up (Ampure XP beads, Beckman Coulter). The resulting cDNA (0.5–1 ng) was tagmented at 55°C for 30 min and final RNA-Seq libraries generated using reagents from the Nextera XT DNA Library Prep Kit (Illumina). Sequencing was performed on a NextSeq 500 machine (Illumina, San Diego, CA) using the NextSeq 500/550 High Output v2 kit (75 cycles).

Nextseq 500 high output v2 kit. All RNA-seq data represent pooled data from at least two distinct biological replicates. Sequencing was performed by the Center for Translational Genomics and Bioinformatics of San Raffaele Scientific Institute, Milan.

Quality of the input reads was assessed using FastQC and read trimming was performed with Trimmomatic to remove adapters and low-quality sequences. Then, trimmed reads were given as input to the STAR aligner software to align them against the human reference genome (GRCh38/hg38) with standard parameters. After that, gene counts were obtained using ‘Subread feature Counts’ with Genocode (v29) gene annotation. Gene expression counts were processed with R using the R/Bioconductor package edgeR, normalizing for library size using trimmed mean of M-values, and correcting p-values using FDR. Lists of differentially expressed genes (FDR < 0.05) were analyzed with clusterProfiler for functional enrichment analysis. Enrichment p-values were corrected for multiple testing using FDR. Gene Set Enrichment Analysis (GSEA) was performed on Gene Ontology and Pathway databases by pre-ranking genes according to log2 (FoldChange) values.

### Flow Cytometric Analysis

Flow cytometric analysis of human TEC was performed by multi-color staining and using the following mAbs: anti-CD326 (EpCam) PE-Vio770 (clone HEA-125), anti-CD45 APC (5B1), anti-HLA-DR, DQ, DP PE (all from Miltenyi Biotec), anti-Ulex-1 FITC (FL 1061, Vector Laboratories, Burlingame, California, USA).

Thymocytes were characterized using a multi-color staining. To discriminate DN, DP, SP4 and SP8 thymocyte subsets, we performed a three-color staining using the following mAbs: CD8 Vio-Blue (clone BW135/80), CD45 APC (5B1) and CD4 PerCP (VIT4) (all mAbs are from Miltenyi Biotec).

Peripheral blood mononuclear cells (PBMCs) were isolated by density gradient centrifugation using Lymphoprep (density: 1.077 g/ml; STEM CELL Technologies, Vancouver, Canada). To isolate total T cells we used the anti-human Pan T cells isolation kit (Miltenyi Biotec). To discriminate CD4^+^ and CD8^+^ T cell subsets we performed a four-color staining using the following mAbs: CD45 APC (clone 5B1), CD3 VioGreen (REA613), CD4 PE-Vio770 (M-T321) and CD8 PE (BW135/80) (all from Miltenyi Biotec).

Surface stainings were performed in PBS with 2% FBS and 0,1% sodium azide for 20 min at 4°C. Cells were acquired using a FACS CantoII (BD Biosciences, San Jose, CA, USA) and analyzed with Flow Jo Software (FLOWJO, LLC, Ashland, OR, USA).

### Senescence Associated - Beta Galactosidase (SA-β-Gal) Analysis

SA-β-gal activity was assessed in TECs, thymocytes and PBMCs. We used a fluorescence-based protocol based on the alkalinization of lysosomes. Cells were plated at a concentration of 2 million cells per well in 12-well plates (CORNING) in pre-warmed fresh RPMI culture medium (CORNING). Cells were treated with a final concentration of 100nM of lysosomal inhibitory drugs bafilomycin A1 (INVIVOGEN, San Diego, California, USA). Bafilomycin A1 is used to neutralize the acidic pH of lysosomes and to allow the detection of SA-β-gal. Cells were incubated for 1 hour at 37°C and 5% CO_2_. Cells were then resuspended in cold PBS (CORNING) containing 5-dodecanoylaminofluorescein di-ß-D-galactopyranoside (C_12_FDG) (ThermoFisher Scientific, Walthman, Massachusetts, USA) a fluorogenic substrate for β-galactosidase. This compound was added at a final concentration of 33uM and cells were incubated for 2 hours at 37°C and 5% CO_2_. C_12_FDG is a substrate which, when hydrolyzed by SA-β-gal, becomes fluorescent and membrane impermeable. Cells were then washed, resuspended in 200 μl PBS and analyzed immediately using a FAS Canto II (BD Bioscences, San Jose, CA, USA) or an EVOS fluorescence microscope (ThermoFisher).

Cells acquired by FACS Canto II (BD Bioscences) were analyzed using the Flow Jo Software (FLOWJO, LLC, Ashland, OR, USA) and the analysis was based on two parameters: forward scatter (FSC) versus side scatter (SSC) region to exclude dead cells and subcellular debris. SA-β-gal activity was expressed as median fluorescence intensity (MFI) on one-parameter histogram displaying FL1, 488, FITC in different cell subpopulations: TECs, thymocytes and PBMCs.

TEC analyzed by the EVOS fluorescence microscope (Leica Microsystems, Rijswijk, The Netherlands) were seeded in Permanox 4-chamber slides (ThermoFisher) at 4 × 10 (3) cells/chamber and allowed to attach for 4 h. After treatment, TECs were fixed with 4% paraformaldehyde (Sigma-Aldrich) in PBS (CORNING) for 4 min. Slides were mounted with Vectashield Fluorescent Mounting Medium (Vector Laboratories, Burlingame, CA) and photographed with the EVOS fluorescence microscope (Leica Microsystems). One-hundred randomly chosen cells per sample were assessed for SA-β-gal positivity density (% of area analyzed).

### RNA Extraction and Gene Expression Analysis

RNA was extracted from TECs after digestion and CD45 cell-depletion, from thymocytes and PBMCS using the RNeasy Micro kit (QIAGEN, Hilden, Germany). Reverse transcription of mRNA was performed with the High Capacity Reverse Transcription Kit (Applied Biosystems, Foster City, CA, USA). Real-time PCR was performed using TaqMan Gene expression Assays (Applied Biosystems) and the EagleTaq Universal Master Mix (Roche, Basel, Switzerland). PCR reactions were performed in MicroAmp^®^Optical 96-well reaction plates (Applied Biosystems) in a final volume of 25 μl and run on the Viia-7 Real-Time PCR machine (Applied Biosystems). Relative quantification of genes was performed with the 2−ΔΔCt method and expressed as fold change relative to the expression of the endogenous control, RPLP0.

### Histology and Morphometric Analysis

Human tissue samples were formalin-fixed and paraffin-embedded. Sections (1.5 μm) were used for routine hematoxylin and eosin (H&E) staining. The following primary antibodies were used: rabbit anti-human Involucrin (Abcam, Cambridge, UK; 1:100; art: microwaves in EDTA buffer pH 8.0; inc: 1 hr at RT), mouse anti-human AIRE (kindly provided by Prof P. Peterson, University of Tartu, Tartu, Estonia; 1:3.000; art: thermostatic bath in EDTA buffer pH 8.0; inc: 1 h at RT), mouse anti-human p16 (BioGenex, Fremont, California, USA; 1:1; art: microwaves in EDTA buffer pH 8.0; inc: 1h at RT). Primary antibodies were incubated with MACH 1™ Universal HRP Polymer Kit (Biocare Medical), and reactions were developed in Biocare’s Betazoid DAB and nuclei counterstained with hematoxylin.

Digital images were acquired by an Olympus XC50 camera mounted on a BX51 microscope (Olympus, Tokyo, Japan) with CellF Imaging software (Soft Imaging System GmbH, Münster, Germany). Morphometric analysis was performed using Olympus Slide Scanner VS120-L100 (Olympus, Tokyo, Japan) to acquire digital images and Image-pro software (Olympus) to analyze them.

### Oxidative Stress Detection

PBMC from DS patients and age matched HDs were also analyzed to assess the level of oxidative stress. To this end, we used two different methods based on the mitochondrial evaluation: MitoTracker Green kit (ThermoFisher) and Tetramethylrhodamine ethyl ester (TMRE) assay.

For mitochondrial staining evaluated by MitoTracker Green kit, used at a final concentration of 300 nM in PBS (CORNING), cells were fully immersed in 1 milliliter of staining solution and incubated on ice for 25 minutes with general agitation. Cells were then washed in PBS for 5 minutes and then to block non-specific binding of the antibodies, cells were submerged in 5% bovine serum albumin (Sigma-Aldrich) for 1 hour at room temperature. Cells were then washed and resuspended in fresh pre-warmed (37°C) PBS (CORNING), acquired using a FACS Canto II (BD Biosciences, San Jose, CA, USA) and analyzed with Flow Jo Software (FLOWJO, LLC). In parallel, TMRE (tetramethylrhodamine, ethyl ester) assay evaluates the mitochondrial membrane potential, a parameter also directly linked to cellular oxidative stress. TMRE (Thermofisher) was dissolved in methanol and used directly adding this compound to PBS at a final concentration of 20nM. Cells were incubated for 30 minutes in the incubator (37°C and 5% CO_2_), then washed in fresh pre-warmed PBS (CORNING) and directly acquired using a FACS Canto II (BD Bioscences, San Jose, CA, USA). Flow cytometric analysis was performed with Flow Jo Software (FLOWJO, LLC).

Oxidative stress was detected also in plasma samples using the OxiSelect *in vitro* ROS Assay Kit (CELL BIOLABS, San Diego, California, USA), following the protocol recommended by the manufacturer.

### Statistical Analyses

Statistical analyses were performed with GraphPad Prism 5.0 (GraphPad Software, San Diego, California, USA). All results are expressed as the mean ± SEM if not stated otherwise. Comparisons between proportions were calculated by using the chi-square test (χ^2^ test) (with continuity correction) as stated in the Figure legends. To assess significance, we used one-way ANOVA with Bonferroni post-correction or two-way ANOVA analysis of variance when specified. We also used two-tailed Mann-Whitney test where specified. *p*-values <0.05 were considered significant.

## Results

### Premature Aging in Thymi of DS Patients

**Figure 1:**
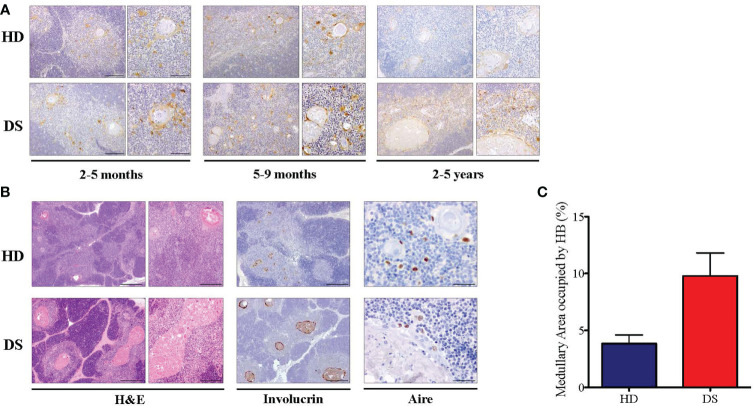
Histological analysis of DS patients thyme. **(A)** Representative histological images of p16 immunohistochemical staining in three different groups of age-matched HD and DS thymic tissue sections (original magnification: 10x = 200 μm left panel – 20x = 100 μm right panel). For each age group, a minimum of 1-3 thymi were analyzed, according to sample availability. **(B)** H&E staining and Involucrin and AIRE immunohistochemical staining of HD and DS thymic samples from a 4 year-old DS patient and a 3 year-old healthy donor (original magnification: H&E 4x = 500 μm left panel -10x = 200 μm right panel; Involucrin 4x = 500 μm: AIRE 40x = 50 μm) (HD sample 12 in **Supplemental Table 2**; DS sample 1 in **Supplemental Table 3**) **(C)** The graph shows the proportion of medullary area occupied by the Hassall’s bodies in thymic samples (HD samples: 2, 7 and 12 in **Supplemental Table 2**; DS samples: 1 and 6 in **Supplemental Table 3**). Median with interquartile range is represented.

In our previous work we showed tissue abnormalities in DS thymi, which are smaller in size and display accelerated kinetics of maturation, as compared to age-matched controls, with signs of premature involution (11). We found an expansion of the medullary area caused by the presence of large cystic involutions positive for involucrin, a marker expressed by terminally differentiated medullary TEC (11). Based on these observations and data reported in literature suggesting immunosenescence in DS patients (10, 12), we evaluated thymic epithelium maturation by immunohistochemical analysis on thymic tissues of DS patients and compared them to age-matched healthy donors. We considered three age groups: 2-5 months, 5-9 months, 2-5 years. As a marker of cellular senescence we analyzed p16, a cyclin-dependent kinase inhibitor that tends to accumulate in cell cycle arrested cells (24). Immunohistochemistry revealed an increased p16 expression associated with large size of Hassall’s bodies in DS patients, as compared to age-matched HDs, which becomes more evident in the oldest patient-group (**Figure 1A**). Moreover, we analyzed the thymic tissue of a 4 year-old DS patient, and the thymic tissue from a 3 year-old healthy donor, evaluating their morphology by H&E staining and by immunohistochemistry the expression of Involucrin which detects terminally differentiated mTECs. In the 4 year-old DS thymus, H&E staining showed a similar cortico-medullary ratio as compared to the healthy control (**Figure 1B**). However, we observed in DS thymic samples an increased size of Hassall’s bodies, identified by the staining with Involucrin, and the amount of medullary area occupied by the Hassall’s bodies in the medulla (**Figure 1C**). Additionally, the 4 year-old DS sample showed a reduced number of AIRE^+^ cells [**Figure 1C** and our previous published data (11)]. No relevant differences were detected by immunohistochemistry in the thymocyte compartment, which showed no alterations in T cell-maturation and expression of developmental markers (TdT, CD3, CD4, CD8).

### Transcriptomic Profile of Human TEC in DS Patients

In order to investigate the mechanisms underlying thymic dysfunction in DS patients, we performed a whole transcriptomic analysis *via* RNA-Seq analysis on sorted CD45^-^ Epcam-enriched hTEC from DS patients and HD. Frozen samples of 3 normal HD and 3 DS patients were analyzed (**Supplemental Table 1**). Differential gene expression (DE) and gene set enrichment analysis (GSEA) provided interesting insights into Epcam-enriched hTEC transcriptome in samples from both HD and DS patients, highlighting distinct transcriptomic profiles (**Supplemental Figure 1A** and **Supplemental Table 5**). First of all, DE analysis confirmed that we were able to substantially enrich TEC in Epcam-positive (Epcam^+^) sorted thymic samples, as genes typically expressed in cTEC and mTEC were significantly more expressed in Epcam^+^ subsets, as compared to Epcam-negative (Epcam^-^) fraction (**Table 1**, **Supplemental Figure 1B**). When we compared Epcam^+^ versus Epcam^-^ samples from both HD and DS patients, enrichment analysis using the REACTOME Pathway and GO (Biological Processes) databases on differentially expressed genes (DEGs), revealed a statistical increased expression of genes involved in extracellular matrix organization, collagen formation and epithelial development (**Supplemental Figure 1C**).

**Table 1:** Differential expression of cTEC-specific genes.

	HD		DS	
	logFC	FDR	logFC	FDR
*Ccl25*	4,5547	2,76E-06	3,5524	0,0002
*Cd74*	1,2122	0,1379	2,4771	0,0004
*Ctsl*	-2,278	7,96E-06	-2,4404	1,68E-06
*Foxn1*	6,7714	5,79E-08	5,6012	3,24E-06
*Ly75*	3,3015	1,87E-05	3,1349	5,44E-05
*Pax1*	5,5748	1,93E-07	3,8492	0,0002
*Prss16*	3,6074	2,3E-05	3,7218	1,26E-05
*Psmb11*	3,5666	0,0537	3,3808	0,0563

Log2-fold change and FDR adjusted p-values (padj) are reported, obtained from the comparison of Epcam^+^ and Epcam^-^ cell subsets within each subject’s group. Positive log2-fold change values indicate upregulation of that specific gene in Epcam^+^ cells.

**Figure 2:**
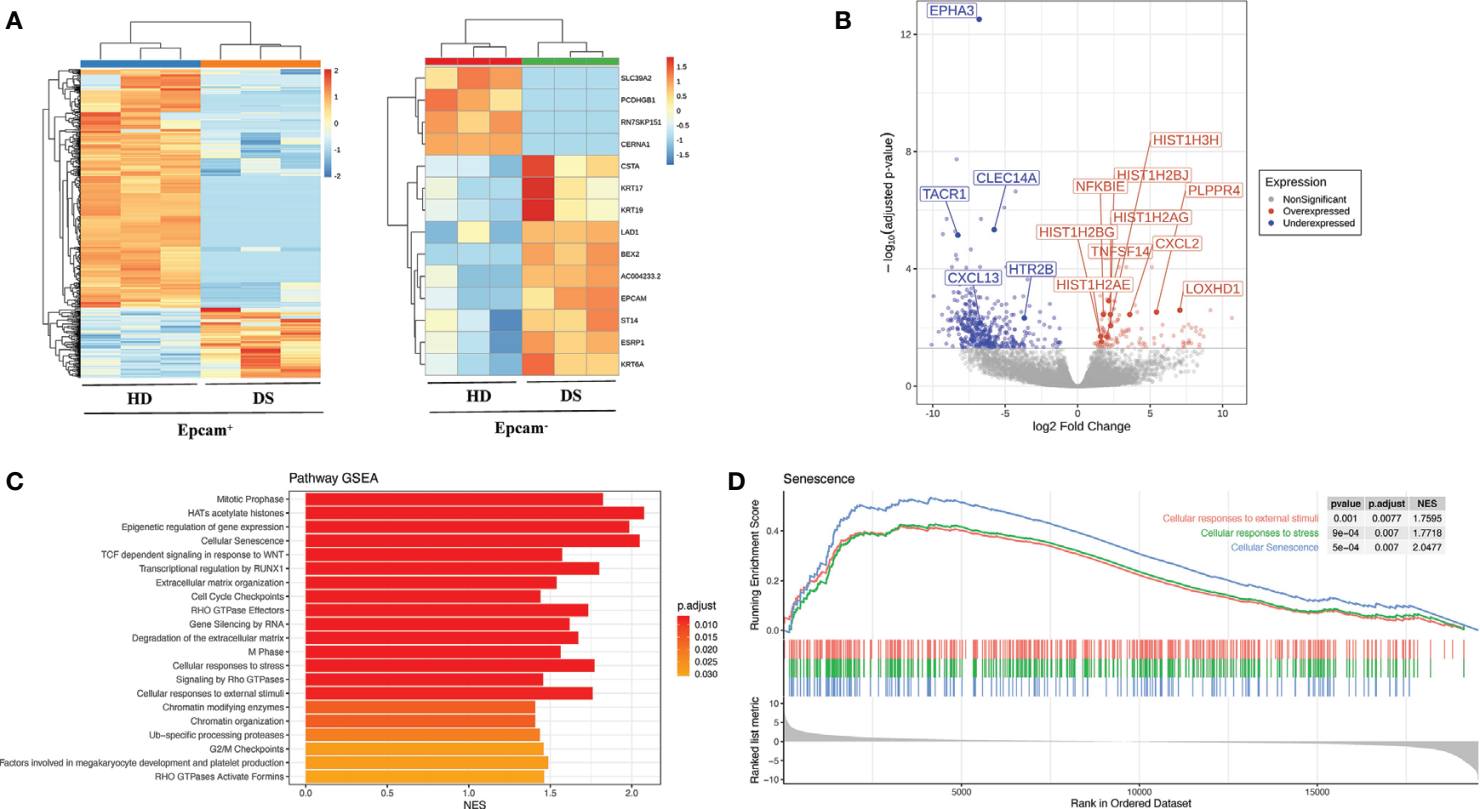
Transcriptomic profile of human TEC in HD and DS patients. **(A)** Heatmaps showing the expression of differentially expressed genes (DEGs) between normal subjects (HD) and DS patients in Epcam+ (left) and Epcam- (right) cell subsets. **(B)** Differentially expressed genes (DEGs) between DS and HD Epcam^+^ cell subsets. Log2 fold change and FDR-adjusted p-values are reported for each gene. Volcano plot showing significant (FDR < 0.05) down- (blue) and up- (red) regulated genes between DS and normal patients in Epcam+ cell subset. Differentially expressed genes of interest are highlighted. **(C)** Significantly enriched terms resulting from the GSEA against the REACTOME Pathway database of genes ranked according to logFC values, resulting from the comparison between DS and normal subjects (HD) in Epcam+ samples. **(D)** Enrichment plot showing the GSEA results of three senescence-related terms on gene pre-ranked based on logFC values resulting from the comparison between DS and normal patients in Epcam^+^ cell subset.

Interestingly, majority of gene expression differences was observed within the Epcam^+^ positive subsets, while Epcam^-^ fraction showed more similarities between patients and healthy subjects, suggesting that main differences between DS patients and normal donors reside in TEC (**Table 2**, **Figure 2A**). Among the most significantly upregulated genes in DS TEC-enriched samples, many transcripts encoding for histones emerged (**Figure 2B**). Furthermore, GSEA revealed several gene sets particularly enriched in DS samples, as compared to HD, suggesting the presence of a disease-specific transcriptomic signature at TEC level. Noteworthy, GSEA results obtained from the comparison between DS and HD Epcam^+^ cell subsets showed a statistically significant enrichment of categories related to cellular senescence, cellular response to stress and epigenetic histone and DNA modifications in DS (**Figure 2C**). In particular, the positive enrichment of cellular senescence, cellular response to stress, and cellular responses to external stimuli terms, strongly suggesting that cells in the Epcam^+^ subset in DS patients present an over-expression of genes involved in the senescence process when compared to normal subjects (**Figure 2D**).

**Table 2:** Differentially expressed genes at FDR < 0.05.

	DS Epcam^+^/DS Epcam^-^	HD Epcam^+^/HD Epcam^-^	DS Epcam^+^/HD Epcam^+^	DS Epcam^-^/HD Epcam^-^
**Upregulated**	2419	1903	125	10
**Downregulated**	2814	2082	424	4
**Total DE genes**	5233	3985	549	14
*(FDR < 0.05)*				

### Thymic Epithelial Cells in DS Show Signs of Senescence

**Figure 3:**
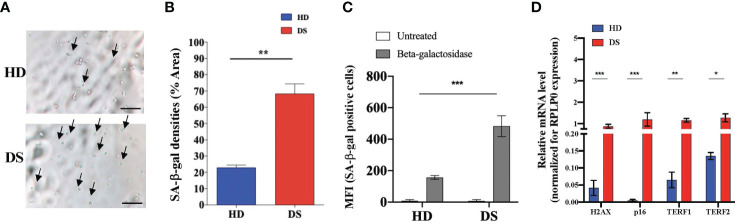
Senescence detection in thymic epithelial cells. **(A–C)** SA-β-gal staining in TEC isolated from DS patients and age-matched HDs. Analyses performed by microscopy **(A, B)** (SA-β-gal^+^ cell counts were normalized per % of Area; scale bar = 100 µm) and flow cytometry **(C)** (HD, n = 9; DS, n = 9; HD 2-5 months: 1,35; 5-9 months: 4,6,9 and 2-5 years 2,7,10 referring to **Supplemental Table 2**; while DS 2-5 months: 2,3,5; 5-9 months: 4,8,9 and 2-5 years 1,6, 10 referring to **Supplemental Table 3**). **(D)** Comparison of mRNA expression of senescence-associated genes (H2AX, p16, TERF1 and TERF2 in thymic tissue from DS patients and age-matched HDs, normalized for the expression of the housekeeping gene RPLP0 (HD, n = 12, sample 13 in **Supplemental Table 2** was excluded from the analyses; DS, n = 9, from sample 1 to sample 9 in **Supplemental Table 2**). Mean ± SEM are represented (Mann-Whitney test; *p-value < 0.05; **p-value < 0.002; ***p-value < 0.0001).

Senescence associated β-galactosidase (SA-β-gal) activity is one the most commonly used biomarkers for senescent cells detection (24). To further characterize TEC senescence in DS patients, SA-β-gal levels were quantified by fluorescent microscopy (**Figures 3A, B**) and flow cytometry (**Figure 3C**) and we reported a significant increase in DS patients as compared to age-matched HDs.

To analyze more in depth the senescence profile of TEC in DS patients we evaluated the expression of four genes normally associated to cellular senescence: H2AX, p16, TERF1 and TERF2 (**Figure 3D**). H2A histone family member X (usually abbreviated as H2AX) is a type of histone protein from the H2A family involved in all senescence mechanisms (25). TERF1 is a negative regulator of telomere length, while TERF2 protects telomers from degradation and fusion and both of them are involved in cellular senescence and in aging processes (26). Real time PCR analysis detected higher expression of all these genes in digested thymic tissue from DS patients after depletion of CD45+ cells, further confirming the establishment of a senescence program in thymic stroma of these patients.

### T-Cell Compartment Is Compromised in DS Patients

**Figure 4:**
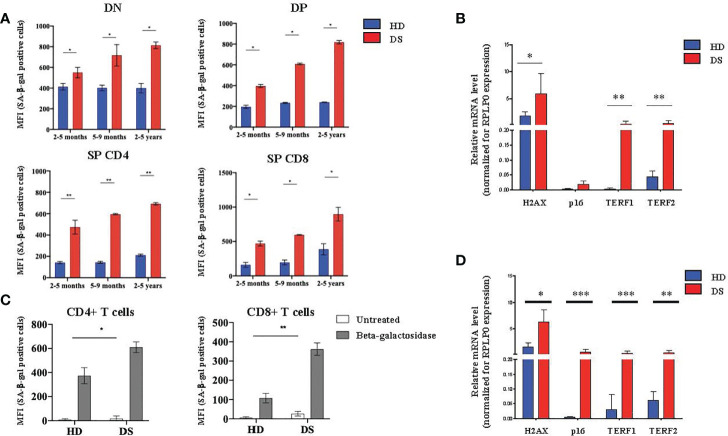
Senescence detection in thymocytes and PBMCs. **(A)** MFI of SA-β-gal positive cells calculated on the gate of DN (CD45^+^CD4^-^ CD8^-^), DP (CD45^+^ CD4^+^ CD8^+^), SP4 (CD45^+^ CD4^+^ CD8^-^) and SP8 (CD45^+^ CD4^-^ CD8^+^) thymocytes. HD and DS samples were divided in three age-groups: 2-5 months, 5-9 months and 2-5 years (2-5 months: HD, n = 3, samples 1, 3, 5 in **Supplemental Table 2**; DS, n = 3, samples 2, 3, 5 in **Supplemental Table 3**. 5-9 months: HD, n = 3, samples 4, 6, 9 in **Supplemental Table 2**; DS, n = 3, samples 4, 8, 9 in **Supplemental Table 3**. 2-5 years: HD, n = 3, samples 2, 7, 10 in **Supplemental Table 2**; DS, n = 3, sample 1, 6, 10 in **Supplemental Table 3**). **(B)** Comparison of mRNA expression of senescence associated genes (H2AX, p16, TER1 and TERF2) in thymocytes isolated from DS patients and age matched HDs, normalized for the expression of the housekeeping gene RPLP0 (HD, n = 12, sample 13 in **Supplemental Table 2** was in excluded from the analyses; DS, n = 9, from sample 1 to sample 9 in **Supplemental Table 3**). **(C)** MFI of SA-β-gal positive CD4^+^ T cells (*left graph*) and CD8^+^ T cells (*right graph*) calculated on the gate of CD45^+^, CD3^+^ T lymphocytes (HD, n = 12, sample 13 in **Supplemental Table 2** was excluded from the analyses; DS, n = 12, from sample 1 to sample 9 in **Supplemental Table 3**). **(D)** Comparison of mRNA expression of senescence-associated genes (H2AX, p16, TER1 and TERF2), in peripheral T cells isolated from DS patients and age-matched HDs, normalized for the expression of the housekeeping gene RPLP0 (HD, n = 12, sample 13 in **Supplemental Table 2** was in excluded from the analyses; DS, n = 9, from sample 1 to sample 9 in **Supplemental Table 3**). Mean ± SEM are represented (Mann-Whitney test; *p-value < 0.05; **p-value < 0.002; ***p-value < 0.0001).

Next, we moved to the lymphoid compartment in which we evaluated SA-β-gal expression in thymocytes isolated from DS patients and HDs. To obtain a better characterization, we divided our samples, both HDs and DS, in three age-groups that represent different stages of thymic maturation and development: 2-5 months, 5-9 months and 2-5 years (27). Flow cytometric analysis was performed in 4 different thymocyte subsets: DN, DP, SP and SP8. In all thymocyte subsets and age-groups analyzed the expression of SA-β-gal was statistically increased in DS patients as compared to age matched HDs. Interestingly, SA-β-gal expression increased more dramatically from 2 months to 5 years in DS patients, as compared to HDs, suggesting an accelerated aging (**Figure 4A**). Similar to TECs, H2AX, TERF1 and TERF2 expression was significantly increased in DS patients as compared to HDs (**Figure 4B**). p16 gene expression in thymocytes from DS patients was increased although it did not reach statistical significance (**Figure 4B**). These results indicate that also the thymocyte compartment in DS patients has increased senescence. To evaluate whether peripheral T cells were also affected, we analyzed β-gal expression in peripheral blood CD4^+^ and CD8^+^ T cells of DS patients and age-matched controls. We noticed an increased SA-β-gal expression in terms of MFI in both CD4^+^ and CD8^+^ T cells in all DS patients as compared to age-matched HDs (**Figure 4C**). Concomitantly, we also found a consistent increased of H2AX, TERF1 and TERF2 expression in total PBMCs from DS patients with data observed in the thymic compartment (**Figure 4D**).

Overall, these data indicate an accelerated senescence establishment affecting thymic epithelial cell compartment as well as thymocytes and leading to the egress of exhausted and senescent lymphocytes to the periphery.

### High Level of Cellular Oxidative Stress in Thymic Compartment and in Peripheral Blood in DS Patients

**Figure 5:**
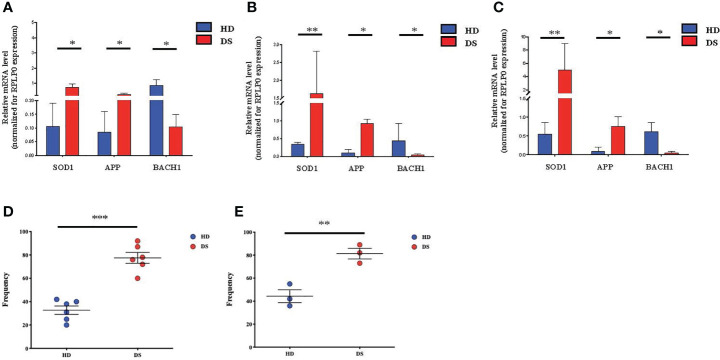
Oxidative stress detection in TEC, thymocytes and PBMCs. **(A–C)** Comparison of mRNA expression of oxidative stress-associated genes (SOD1, APP, BACH1), in TEC **(A)**, thymocytes **(B)** and PBMCs **(C)** isolated from DS patients and age-matched HDs, normalized for the expression of the housekeeping gene RPLP0 (TEC and thymocytes: HD, n = 12, sample 13 in **Supplemental Table 2** was excluded from the analyses; DS, n = 9, from sample 1 to sample 9 in **Supplemental Table 3**. PBMCs: HD, n = 12, sample 13 in **Supplemental Table 2** was excluded from the analyses; DS, n = 9, from sample 1 to sample 9 in **Supplemental Table 3**). **(D)** Frequency of mitochondria function and number in PBMCs evaluated on the gate of total CD45^+^ CD3^+^ T lymphocytes isolated from DS patients and age-matched HDs (HD, n = 6, samples 2, 7, 9, 10, 11,12 in **Supplemental Table 2**; DS, n = 6, samples 1, 6, 9, 10, 11, 12 in **Supplemental Table 3**). **(E)** Analysis of mitochondrial membrane potential, expressed as frequency and calculated on the gate of total CD45^+^ CD3^+^ T lymphocytes isolated from DS patients and age-matched HDs (HD, n = 3, samples 2, 7, 11 in **Supplemental Table 2**; DS, n = 3, samples 10, 11, 12 in **Supplemental Table 3**). Mean ± SEM are represented (Mann-Whitney test; *p-value < 0.05; **p-value < 0.002; ***p-value < 0.0001).

Growing evidence supports the close link between cellular senescence and oxidative stress (28–30), due to the excessive production of reactive oxygen species (ROS), which leads to the accumulation of oxidative damage and induces changes in molecules, cells and tissues (31). ROS are produced by several endogenous and exogenous processes and their negative effects are neutralized by antioxidant defenses. Oxidative stress occurs from the imbalance between ROS production and antioxidant defenses (30). Based on these observations and on our previous results, we investigated the correlation among senescence, oxidative stress and mitochondrial dysfunction in DS (**Figure 5**). Indeed, mitochondria are deeply involved in the production of reactive oxygen species and are also very susceptible to oxidative stress. Oxidative stress can induce apoptotic death, and mitochondria have a central role in this and other types of apoptosis (32). To investigate whether the oxidative stress might have a role in accelerated senescence in DS patients, we analyzed the expression of oxidative stress promoter Superoxide Dismutase (SOD1), Amyloid Beta Precursor Protein-(APP), which is induced by oxidative stress (32), and BACH1, an antioxidant gene. The analysis was performed on TEC (**Figure 5A**), total thymocytes (**Figure 5B**) and PBMCs (**Figure 5C**) isolated from DS patients and HDs. Our results indicate an increased expression of SOD1 and APP genes in DS patients as compared to age-matched HDs in all three different cell types (**Figures 5A–C**); conversely the antioxidant gene BACH1 resulted significantly decreased in DS patients as compared to age-matched HDs in TEC, thymocytes and PBMCs (**Figures 5A–C**, respectively).

We then evaluated mitochondria functionality, by quantifying their number and their surface membrane potential. The analysis was performed on total T lymphocytes isolated from DS patients and age-matched HDs. In both flow cytometric analyses performed using two different methods, Mitotracker (**Figure 5D**) and TMRE analysis (**Figure 5E**) to evaluate mitochondria and label active mitochondria respectively, we noticed an alteration of cellular organelles in DS patients’ cells. The frequency of mitochondria was significantly increased as compared to age-matched HDs, while analysis of mitochondria membrane potential showed an increased frequency of active mitochondria in DS patients as compared to age-matched HDs.

Altogether, our results indicate an increased level of oxidative stress in TEC, thymocytes and PBMCs isolated from DS patients in terms of increased ROS production associated to a decreased antioxidant function and increased mitochondria numbers and membrane potential.

## Discussion

DS patients present with specific conditions associated with earlier aging as compared to general population, including premature skin wrinkling, greying of hair, hypogonadism, early menopause, hypothyroidism, Alzheimer’s disease and declining immune function (12). Premature aging in DS is atypical and segmental, involving some but not all organs and tissues, particularly the brain and the immune system (12). Several studies have reported alterations of adaptive immune system including defects affecting thymocytes and mature peripheral lymphocytes (9, 12). These defects result in a high predisposition to develop recurrent infections, especially of the respiratory tract, autoimmune diseases and leukemia’s or other lymphoreticular malignancies (8, 9, 13). The recent description of DS individuals showing increased risk of dying from COVID-19 infection has further highlighted their defective and senescent immune system (33). Several are the mechanisms underlying immune perturbation. Various observations, including the fact that chronic diseases associate with aging, point to an accelerated senescence process as the major culprit in DS (12, 13). Consistently, DS patients’ immunological profile is suggestive of precocious immunosenescence, characterized by earlier thymic involution with low thymic output and decreased number of naïve CD4^+^ and CD8^+^ T lymphocyte (10–13). The lack of data on TEC and thymocytes vs PB obtained from the same patient and how these immune parameters change over time, prompted us to further evaluate the contribution of various cellular players (epithelial vs thymocytes and lymphocytes) on DS pathogenesis focusing on senescence process. We speculated that accelerated senescence both in TEC and thymocytes might act as disruptive element of central tolerance homeostasis (12–14). Data from the *TS65Dn* mouse model show increased oxidative stress and reduced cytokine signaling thus pointing to the major role of premature senescence in the pathogenesis of the disease (8, 9). More recently, the demonstration of a perturbed Nrf2 signaling, a pathway required to prevent oxidative damage in DS human fibroblasts and *Dp16* mouse embryonic fibroblasts (34) has provided an additional layer of complexity to DS pathogenesis and focused the attention to the effect of trisomy on the mitochondrial activity. Given these data, we analyzed DS thymic specimens obtained from three different cohort of pediatric patients, confirming the already known signs of thymic involution characterized by a general reduction of thymic size and premature increase of medulla mainly due to enlargement of Hassall’s bodies including highly enriched involucrin positive cells and cystic involutions (10, 11, 18). First, we tested the expression of p16, a marker accumulating in senescent cells and indicating the accelerated maturation process leading to a premature thymic involution. Immunohistochemistry revealed the dramatic increase of p16 in all thymi analyzed, irrespective of the age. Transcriptomic profile was performed on the total epithelial component obtained after depletion of CD45^+^ cells and enriched in TEC cells based on the Epcam surface expression. Noteworthy, for technical reasons, we could not isolate mTEC versus cTEC subpopulations and the different ratio between these cell types might contribute to gene expression changes. However differential gene expression analysis based on TEC subset specific genes did not reveal significant differences between HD and DS Encamp+ cells (data not shown). Overall, the comparison between Epcam^+^ and Epcam^-^ cell fraction transcriptomic profiles highlighted the effect of trisomy on Epcam^+^ subset transcriptomic modulation. Consistently, within this cellular subset in DS we found the majority of differences in gene expression, further supporting the hypothesis of immune dysregulation as an effect of perturbed central tolerance mechanism (18). Conversely to the assumption that perturbation of AIRE and promiscuous gene expression is the main driver of immune dysregulation in DS sustained by some authors (18), here we evaluated whether other players may cause the altered thymic function. Among the genes overexpressed in DS Epcam^+^-enriched subset, we found an increased expression of gene sets involved in cellular response to stress and in senescence process. Many transcripts encoding for histones emerged among the most significantly overexpressed genes in DS TEC-enriched sample corroborating the hypothesis that a leverage of epigenetic mechanisms may counteract immune dysregulation induced by trisomy 21 as originally speculated by Moreira-Filho et al. (20).

These data are in line with the peculiar feature of senescent cells showing distension of pericentromeric satellite sequences and profound changes in epigenome organization (35). To further assess the establishment of the senescent process in TEC, epithelial cells were stained with SA-β-galactosidase showing an increased frequency of positive cells in the area analyzed together with augmented MFI expression detected by flow cytometry. These cells express higher level of H2AX, an indicator of DNA damage response activation, and increased expression of senescent markers p16 and TERF1/TERF2, two proteins of the sheltering complex involved in the maintenance of capping function and telomere length (36). Increased expression of SA-β-gal, p16 and telomerase markers were also found in thymocytes at various differentiation stages irrespectively to the age, confirming that both component epithelial and thymocytes undergo accelerated senescent process.

Extensive studies have shown the interplay between oxidative stress, aging and senescence (35). Reactive oxygen species (ROS) act as signaling molecules that may be detrimental for the cells if not counteracted by antioxidant molecules such as superoxide dismutase (SOD1), glutathione peroxidase and other molecules. In thymocytes and PBMC of DS patients, analysis of SOD1 showed increased levels of expression together with Amyloid Beta Precursor Protein (APP), a molecule mapping on chromosome 21 and induced by oxidative stress (37). BACH1, the transcription regulator protein binding to the promoter of genes containing antioxidant response elements (ARE) to repress cellular antioxidant responses (38), was found decreased in DS patients as compared to healthy donors. All these findings confirmed the increased stress response observed by RNA-seq analysis. Increased oxidative stress has been linked to the accumulation of dysfunctional mitochondria (39), which in turn play a relevant role in triggering senescent process (40). To assess mitochondria function in DS T cells, we measured the frequency of activated mitochondria by measuring membrane potential by TMRE assay in circulating lymphocytes. We found an increased number of mitochondria with increased membrane potential in DS indicating a perturbation of mitochondrial fitness. This perturbed homeostasis further sustains the increased levels of ROS contributing to cellular damage.

Overall, our data provide evidence that immune dysregulation in DS is caused by multiple dysfunctional layers acting in both thymic epithelial cells and thymocytes. Telomere damage, increased senescent process induced by mitochondrial dysfunction and accumulation of ROS all contribute to premature thymic involution with consequent alterations in thymocytes maturation kinetics and egression of exhausted lymphocytes. These data are supported by changes in Epcam-enriched TEC gene expression profile suggesting a disease specific transcriptomic signature. Further dissection of the mechanisms underlying the senescent process in DS, possibly using single cell-based analysis, will be instrumental to envisage novel therapies slowing disease progression in DS patients.

## Data Availability Statement

The datasets presented in this study can be found in online repositories. The names of the repository/repositories and accession number(s) can be found in the article/**Supplementary Material**.

## Ethics Statement

The studies involving human participants were reviewed and approved by San Raffaele Ethics Committee. Written informed consent to participate in this study was provided by the participants’ legal guardian/next of kin.

## Author Contributions

GM, FF, and IB performed experiments, analyzed data and wrote the manuscript. MG performed RNA-Seq profile. StB and IM analyzed data of RNA-Seq TEC. EF performed histological analyses on human tissue samples; ACo performed senescence experiments in **Figures 3A–C** and **4A–C** and interpreted data. DM, MC, SoB, VD’O, AG, DA, CC, and ACa provided samples of patients and healthy donors. RM designed and supervised experiments. AV and MB designed research experiments, supervised the study and wrote the manuscript. All authors contributed to the article and approved the submitted version.

## Funding

This work was supported by the Italian Telethon Foundation (Telethon Core Grant TGT16F03) to MB and by the Intramural Research Program of the DIR, NIAID, NIH. Ricerca Corrente from Childrens’ Hospital Bambino Gesù, Rome, Italy (201702P003966) to CC. This work in RM lab was supported by Telethon (TIGET grant E5) and a Career Development Award from the Human Frontier Science Program. RM is a New York Stem Cell Foundation - Robertson Investigator. AC was supported by the Lady Tata Memorial Trust International Award for Research in Leukaemia 2020-2021.

## Conflict of Interest

The authors declare that the research was conducted in the absence of any commercial or financial relationships that could be construed as a potential conflict of interest.
