# Supplementary material for: Premature Senescence and Increased Oxidative Stress in the Thymus of Down Syndrome Patients
Source: Front Immunol. 2021 Jun 1;12:669893. doi: 10.3389/fimmu.2021.669893 (PMC8204718; doi:10.3389/fimmu.2021.669893)
Supplement: Supplementary file 1 [file DataSheet_1.docx]

**Supplemental material**

**Supplemental Figures**

**Supplemental Figure 1. Transcriptomic profile of human TEC in HD and DS patients**

**(A)** Heatmaps showing the expression of differentially expressed genes (DEGs) between Epcam^+^ and Epcam^-^ cell subsets in normal subjects (*left*) and DS patients (*right*).

**(B)** LogFC values resulting from the comparison between Epcam^+^ and Epcam^-^ cell subsets in normal subjects and DS patients of TEC-specific genes.

**(C)** Top 10 and 5 categories resulting from the enrichment analysis on genes differentially expressed genes between normal subjects and DS patients in Epcam^+^ and Epcam^-^ cell subsets against the REACTOME Pathway and GO (Biological Processes) databases, respectively.

**Supplemental Figure 2. ROS detection in plasma samples in healthy donors (HD) and DS patients.**

**Supplemental Tables**

**Supplemental Table 1. Age of patients whose hTEC were analyzed with RNASeq and number of sorted cells**

| **Patient**  **code** | **Age** | **Nb of sorted cells**  **Epcam^+^ Epcam^-^** | |
| --- | --- | --- | --- |
| **HD-A** | 4 mo | 2550 | 18000 |
| **HD-B** | 4.5 yrs | 18000 | 46000 |
| **HD-C** | 10.2 mo | 11340 | 47930 |
| **DS-A** | 4.5 mo | 1345 | 28000 |
| **DS-B** | 4 mo | 1700 | 22100 |
| **DS-C** | 11 mo | 7400 | 41400 |

**Supplemental Table 2. Healthy Donors included in the manuscript.**

We received and analyzed thymic tissue recovered from all HDs listed in the table.

mo, months; yrs, years.

| **HD** | **Age at sampling** | **Sex** |
| --- | --- | --- |
| **1** | 3 mo | M |
| **2** | 4 yrs | F |
| **3** | 3 mo | M |
| **4** | 5 mo | F |
| **5** | 2 mo | F |
| **6** | 6 mo | M |
| **7** | 2 yrs | M |
| **8** | 4 mo | F |
| **9** | 1 yr | F |
| **10** | 6 yrs | F |
| **11** | 6 yrs | F |
| **12** | 3 yrs | F |
| **13** | 12 yrs | M |

**Supplemental Table 3. DS Patients included in the manuscript.**

*Analysis of thymic tissue. ARDS, acute respiratory distress syndrome; mo, months; yrs, years.

| **DS** | **Age at sampling** | **Sex** |
| --- | --- | --- |
| **1*** | 4 yrs | F |
| **2*** | 4 mo | M |
| **3*** | 3 mo | F |
| **4*** | 6 mo | F |
| **5*** | 2 mo | F |
| **6*** | 2 yrs | F |
| **7*** | 3 mo | M |
| **8*** | 5 mo | M |
| **9*** | 1 yr | M |
| **10*** | 6 yrs | F |
| **11** | 4 yrs | M |
| **12** | 2 yrs | M |

**Supplemental Table 4. Number of patients evaluated for TECs, thymocyte and peripheral blood analysis.**

|  |  | **HD** | | | **DS** | | |
| --- | --- | --- | --- | --- | --- | --- | --- |
|  |  | TEC | Thymocytes | PB | TEC | Thymocytes | PB |
| **AGE GROUPS** | 2-5 months | 3 | 3 | 4 | 3 | 3 | 4 |
|  | 5-9 months | 3 | 3 | 4 | 3 | 3 | 4 |
|  | 2-5 years | 3 | 3 | 4 | 3 | 3 | 4 |
