# Supplementary figures and images for: Premature Senescence and Increased Oxidative Stress in the Thymus of Down Syndrome Patients
Source: Front Immunol. 2021 Jun 1;12:669893. doi: 10.3389/fimmu.2021.669893 (PMC8204718; doi:10.3389/fimmu.2021.669893)

Suppl Figure 1

A

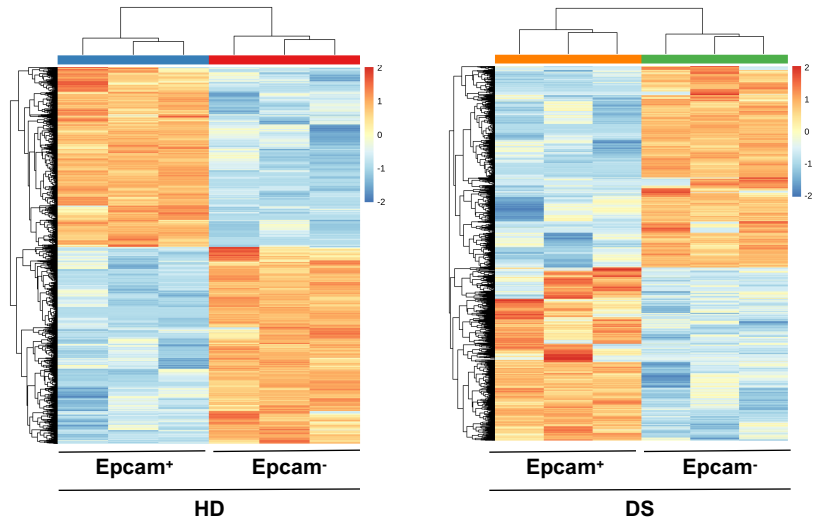

B

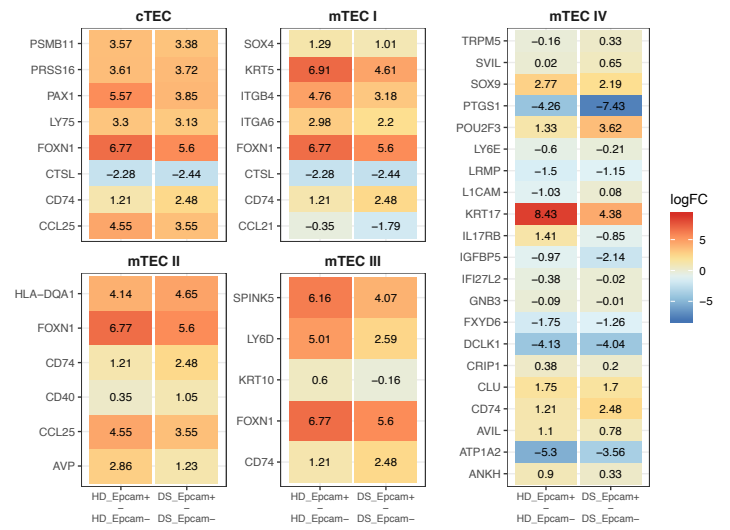

C

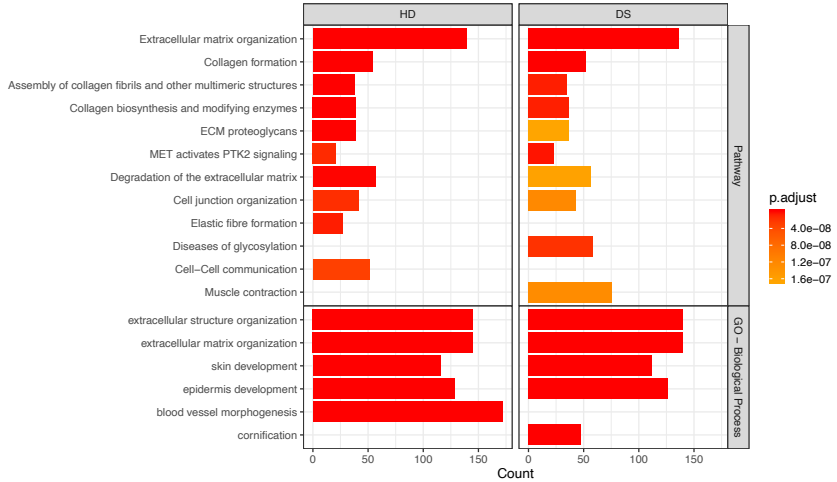

Supplement: Supplementary file 3 [file Image_1.pdf]

Suppl Figure 2

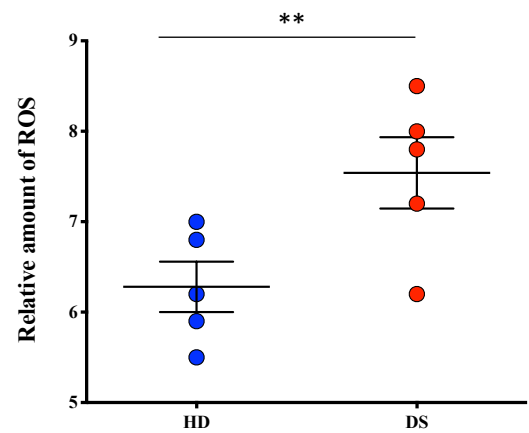

Supplement: Supplementary file 4 [file Image_2.pdf]
